# Finding Constellations in Chemical Space Through Core Analysis

**DOI:** 10.3389/fchem.2019.00510

**Published:** 2019-07-16

**Authors:** J. Jesús Naveja, José L. Medina-Franco

**Affiliations:** ^1^PECEM, School of Medicine, Universidad Nacional Autónoma de México, Mexico City, Mexico; ^2^Department of Pharmacy, School of Chemistry, Universidad Nacional Autónoma de México, Mexico City, Mexico

**Keywords:** analog series, data visualization, descriptor, scaffold, structure-property relationships

## Abstract

Herein we introduce the constellation plots as a general approach that merges different and complementary molecular representations to enhance the information contained in a visual representation and analysis of chemical space. The method is based on a combination of a sub-structure based representation and classification of compounds with a “classical” coordinate-based representation of chemical space. A distinctive outcome of the method is that organizing the compounds in analog series leads to the formation of groups of molecules, aka “constellations” in chemical space. The novel approach is general and can be used to rapidly identify, for instance, insightful and “bright” Structure-Activity Relationships (StARs) in chemical space that are easy to interpret. This kind of analysis is expected to be especially useful for lead identification in large datasets of unannotated molecules, such as those obtained through high-throughput screening. We demonstrate the application of the method using two datasets of focused inhibitors designed against DNMTs and AKT1.

## Introduction

The concept of chemical space is broadly used in drug discovery because of its multiple potential applications; for instance, in library design, compound or dataset classification, compound selection, exploration of structure-activity relationships (SAR), and navigation though structure-property relationships (SPR) in general. However, a precise unique definition of chemical space is not simple. An even more challenging task is the visual representation of this subjective concept.

Chemical space is usually defined as the set of all possible organic compounds (Lipinski and Hopkins, 2004). It is widely recognized that the virtual chemical space is more than astronomically large, as not even all atoms in the universe would suffice to synthesize a single molecule from each of all the 10^63^ possible organic compounds of a size up to 30 atoms (Clayden et al., 2012). Nevertheless, massive efforts have been undertaken to enumerate billions of hypothetical organic compounds, thus allowing large virtual screening campaigns to take place (Reymond, 2015; Lyu et al., 2019).

Along with the increasing size of the mapped chemical space, the interest of applying cartographic methods to visualize the space has expanded (Oprea and Gottfries, 2001). As a result, numerous visualization and conceptualization approaches into chemical space have emerged (Larsson et al., 2007; Osolodkin et al., 2015; Naveja and Medina-Franco, 2017). A cornerstone and key aspect of all proposed methods is the molecular representation and parameters used to define the space where the compounds will reside. Chemical space visualizations have to reduce the dimensionality of the problem of comparing molecular structures, which can be done through algorithms such as principal components analysis and t-distributed stochastic neighbor embedding (see Osolodkin et al., 2015).

In most chemical space approaches, it is desirable that chemical analogs are closer to each other than unrelated and dissimilar molecules since this allows machine learning methods to identify clusters of structurally-related molecules (Medina-Franco et al., 2008; Naveja and Medina-Franco, 2015; Naveja et al., 2016, 2018a). In addition, clustering analog series would allow, at least in principle, to map SAR/SPR into that space. However, due to the vast amplitude of the chemical space and the inevitable loss of information with an initially large space projected into lower dimensions, it is expected that non-analog compounds will end up in the same cluster. Also, when many points in the chemical space are considered at once, visualizations become harder to interpret. To address this issue, approaches such as virtual reality have emerged (Probst and Reymond, 2018).

In parallel to such chemical space approaches based on coordinates, scaffold analysis is a more consistent and chemically-intuitive approach for exploring and identifying collections of analogs (Hu et al., 2011). Ever since the pioneering work by Bemis and Murcko (1996), computational identification of chemical scaffolds has been refined. In this line, Stumpfe et al. (2016) recently introduced the analog series-based scaffold (ASBS), a revolutionary scaffold concept that is more flexible and chemically sound than its predecessors. In fact, the ASBS has proven to yield more biologically meaningful structure-activity/property relationships (SA/PR) than other scaffold definitions (Dimova et al., 2016; Kunimoto et al., 2017; Bajorath, 2018; Dimova and Bajorath, 2018).

Although the chemical space of single analog series can be effectively explored and used, for instance, to guide lead optimization programmes (Vogt et al., 2018), methods for analyzing the relationship among scaffolds of different analog series remain to be explored. Of note, a difficulty in this regard emerges as analog-series based scaffolds tend not to be as consistent as Bemis-Murcko scaffolds, since they result from the retrospective analysis of analog series (Bajorath, 2018). Accordingly, a core framework inspired in the design of the ASBS avoids the shortcoming of inconsistency by allowing molecules to be annotated with more than one putative core (Naveja et al., Submitted). Hence, large libraries containing analogs can be condensed into fewer cores. In this way, SA/PR can be preferentially analyzed for the most explored regions of the chemical space: analog series.

Herein, we present a general methodology for applying the putative core framework to produce more concise and meaningful representations of the chemical space. To our understanding, this is the first method designed for charting multiple analog series into a coordinate-based chemical space, thus combining in a single plot two general and useful approaches of molecular representation and mapping. Of note, since within this framework cores may share analogs (i.e., analog series are allowed to share compounds), such cores can be connected, thus resembling constellations in the chemical space. Therefore, we termed the resulting graphics “constellation plots.” As it will be discussed, activity data (or any property of interest) can be mapped into the constellation plot allowing to explore SA/PRs in the space and quickly identify interesting regions in the space. The rest of this methodological paper is organized as follows: first, the concept scheme is presented and the formalism explained through a toy example; thereafter, two case studies using exemplary datasets are presented; finally, we discuss the conclusions and perspectives of this novel approach for combining the scaffold and the chemical space concepts.

## Methods

### Datasets Used in the Examples

For illustrating the application of constellation plots in two different context of analysis, we used two benchmark datasets that have been previously explored with other analysis approaches. One set was a group of 827 AKT1 inhibitors extracted and curated from ChEMBL (Gaulton et al., 2017; Naveja et al., 2018b). The second dataset was a collection of 286 compounds tested as inhibitors of DNMT (DNA methyltransferases). This second data set was integrated from multiple sources of information as described in Naveja and Medina-Franco (2018). Since this dataset integrates qualitative (such as those containing crystallographic data) and quantitative databases (such as those containing experimental determination of inhibition curves), for this dataset, we use a categorical classification of activity in “active” or “inactive.” The files of the two datasets are included as Supplementary Information.

### Chemical Space and Analog Series

**Figure 1:**
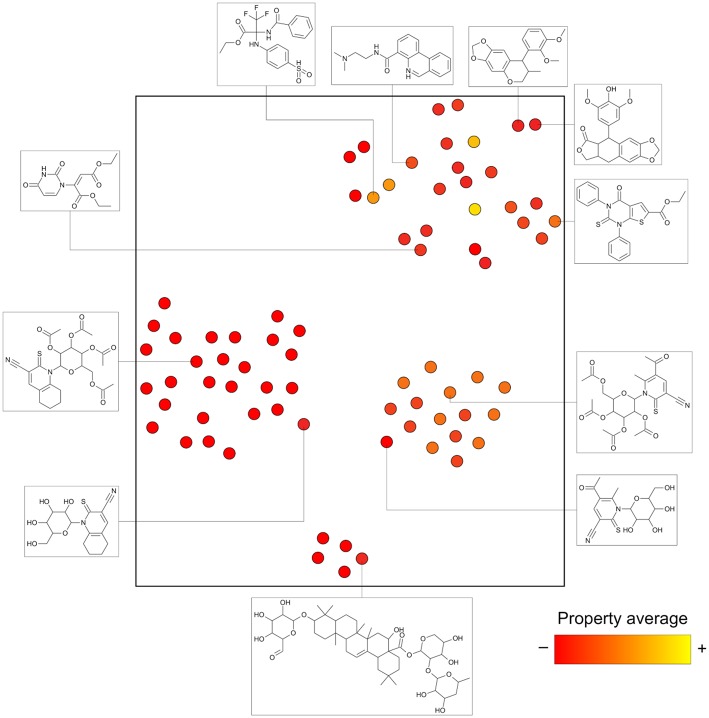
A hypothetical example of a typical chemical space representation based on coordinates. The axes represent the chemical space and have abstract meanings regarding a combination of descriptors. In this case, *t*-SNE using Morgan fingerprints was applied. Every dot represents a single molecule. In activity landscape modeling, color is used to indicate a property (potency in a particular biological endpoint).

As mentioned above, constellation plots fuse two ligand-based concepts: chemical space and core analysis. Standard chemical space analysis is carried out by computing descriptors for a collection of molecules (e.g., physicochemical properties and/or structural features) and then applying dimensionality reduction approaches (Rosén et al., 2009; Osolodkin et al., 2015; González-Medina et al., 2016; Prieto-Martínez et al., 2016; Naveja and Medina-Franco, 2017; Borrel et al., 2018). As a result, every data point represents a single molecule (see Figure 1). This can render many visualizations hard to read and analyze by the naked eye. Furthermore, the numerous descriptors used are combined, such that every axis in the visualization turns out to have a quite abstract meaning. Herein, for the purpose of charting chemical space, *t*-distributed stochastic neighbor embedding (*t*-SNE) is used. This methodology reduces the number of data points in the center of the map as compared to other approaches and has been used successfully in chemical space charting (Maaten and Hinton, 2008; Lewis et al., 2015). However, other coordinate-based representations of chemical space can be used in this general approach.

In contrast to chemical space, standard scaffold and analog series analysis aims toward a clear and consistent picture of the relationships among compounds. For instance, a scaffold is a substructure shared by all compounds annotated with it. A state-of-the-art approach for defining analog series-based scaffolds was proposed by Stumpfe et al. (2016). They have reasoned that for a scaffold to be relevant in medicinal chemistry, it should not only be a substructure of a molecule, but it also has to comply with three additional criteria: (i) be a major component of the whole molecule, (ii) be derived from the molecule through retrosynthetic rules, and (iii) summarize an analog series in a particular dataset. A number of computational approaches for obtaining ASBS have been proposed (Dimova et al., 2016; Stumpfe et al., 2016; Bajorath, 2018; Naveja et al., 2019). Within these approaches, an analog series is defined as a subnetwork connected by matched molecular pairs (MMPs) (Griffen et al., 2011).

**Figure 2:**
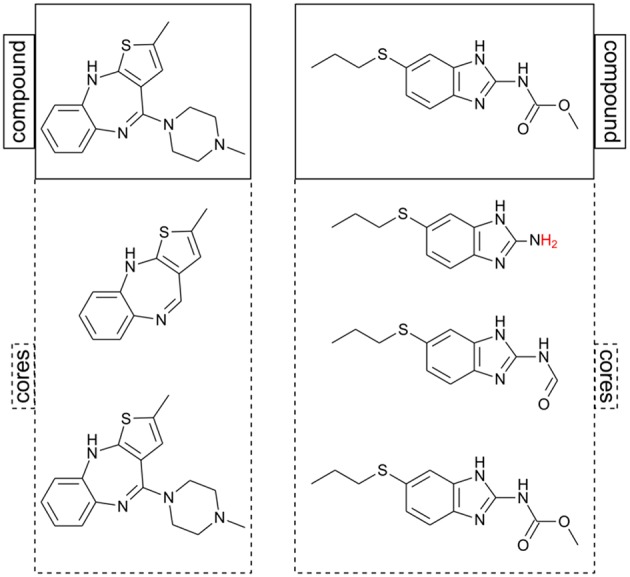
Two examples of putative cores computed for two molecules. Note that in this approach the same chemical structure can be its own core (structures at the bottom). After RECAP fragmentation, hydrogens are added to the core structure to avoid invalid valence (marked in red).

Chemical space analysis of individual analog series has been carried out to measure progression in lead optimization and saturation of analog series (Kunimoto et al., 2018; Vogt et al., 2018; Yonchev et al., 2018). Nevertheless, the fact that assumption (iii) makes analog series inconsistent in as much as the scaffold definition is dependent on the dataset used (Bajorath, 2018) is a limitation for the exploration of chemical space of multiple analog series at once. In a recent study (Naveja et al., Submitted), we discussed that by removing assumption (iii) two effects take place: first, every molecule is allowed to be annotated to more than a single core (equivalent to the term “scaffold”); and second, complete consistency is achieved as no core annotations are ever omitted for any molecule (see Figure 2). It is within this general core framework that we propose using constellation plots.

### Summarizing Analog Series Information in a Dataset Within the General Core Framework

**Figure 3:**
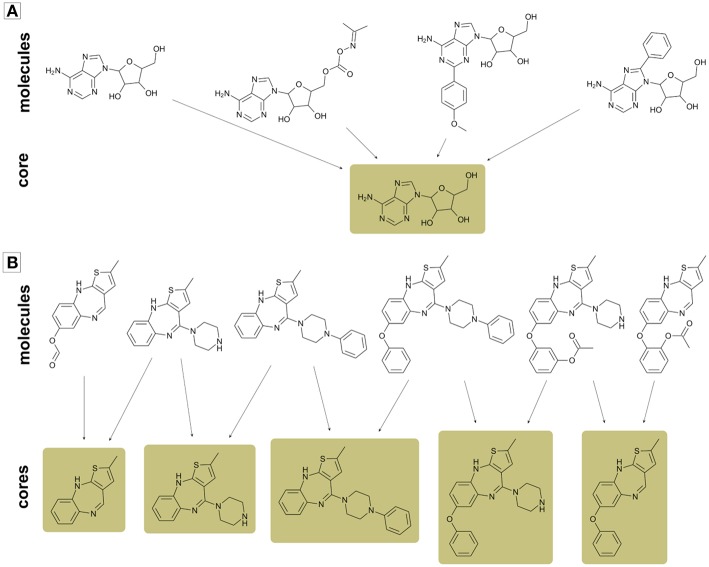
Examples of two analog series with multiple compounds and cores. **(A)** Analog series that can be summarized in a single core; **(B)** Analog series formed by multiple cores. In case **(B)** a single core is not enough for summarizing all information in an analog series.

Since the general core framework can assign multiple cores to single molecules, a useful step prior to mapping cores in the chemical space would be summarizing analog series in the smallest number of cores possible. As illustrated in Figure 3, in some instances it is possible to summarize a whole analog series in a single core structure, while in other cases this cannot be done without loss of information. Hence, for avoiding such situations, we did not discard cores unless only one compound mapped to it. Furthermore, if two or more cores mapped to exactly the same compounds, then only the largest core was kept and the others were disregarded from the analysis.

### Constellation Plots

**Figure 4:**
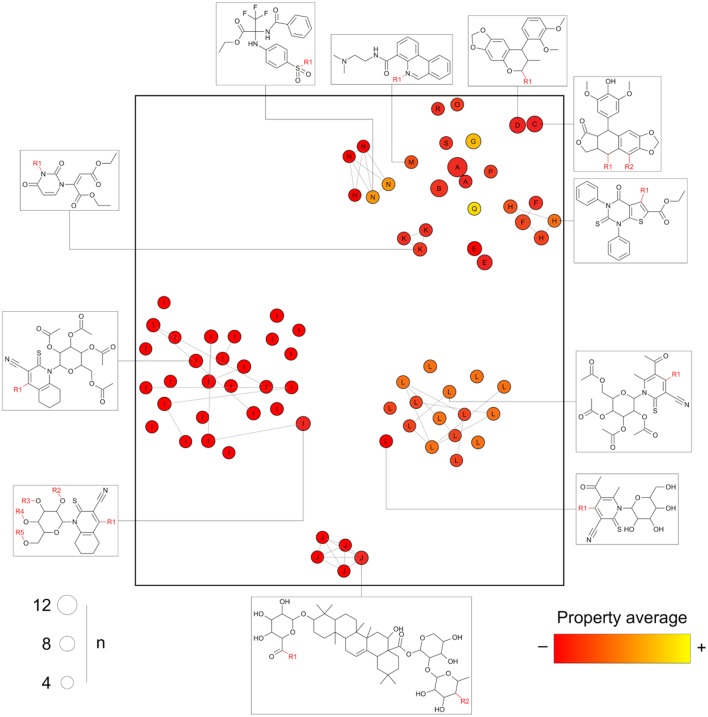
Schematic representation of a general form of a constellation plot. Every circle in the plot represents a core; the axes comprise the coordinates of the chemical structure of the core projected into a 2D representation of the chemical space as computed by any of the standard approaches (e.g., generated using continuous properties or molecular fingerprints and applying *t*-SNE or principal components analysis); the size of the circles indicates the “n” number of compounds annotated to a given core; connected circles are cores sharing compounds; the labels indicate the analog series every point belongs to; the color scale represents the average of a property/activity of the compounds mapping to the core.

After processing a collection of compounds under the general core framework, information is obtained in multiple regards, namely: (a) the chemical structure of every core; (b) the sets of molecules mapping to each core; (c) the molecules annotated to multiple cores; and (d) the analog series to which each compound and core are annotated. We propose a visualization methodology summarizing these four dimensions in a single graphic: the constellation plot that is schematically illustrated in Figure 4.

Essentially, in a constellation plot, the chemical structure of representative cores in a database (for example, those annotated with a predefined minimum number of compounds) is used to find descriptors and map them into a chemical space as if they were single molecules. The size of the circles is used to represent the relative number of compounds annotated to each core. Cores sharing compounds are connected by lines forming “constellations” in the chemical space. Every circle is labeled with an identifier for the analog series to which each core belongs. Additionally, a color scale can be used to represent an average of a given property or activity of the compounds annotated with each core, thereby turning constellation plots useful for activity landscape modeling (Waddell and Medina-Franco, 2012). Of note, the activity can be, for instance, measured for a single molecular target. However, the property could also be a representative measure of the selectivity or promiscuity profile of all the compounds sharing a core across multiple biological endpoints (see section Conclusions and Perspectives).

Figure 4, as opposed to Figure 1, is able to summarize a larger number of compounds than points depicted and contains information about actual analogs. For instance, analog series I, J, and L form separate clusters, but the cluster top right has multiple chemotypes of distinct analog series. This could not be inferred from clustering algorithms applied to the chemical space information only.

### Implementation

All scripts required for producing the data herein reported use free Python code and are made freely available in Supplementary Information. RDkit was used for computing fingerprints and manipulation of chemical structures (http://www.rdkit.org). Scikit-learn was used for computing *t*-SNE (Pedregosa et al., 2011).

## Results and Discussion

The construction of constellations plots and exemplary applications are illustrated with two case studies of general interest in drug discovery. As mentioned in the section Methods, the first example consists of a dataset of 827 AKT1 inhibitors obtained from ChEMBL (Gaulton et al., 2017) and cheminformatically described in Naveja et al. (2018b). The second example employs a data set of 286 DNA methyltransferase (DNMT) inhibitors obtained from the integration of several databases as described in Naveja and Medina-Franco (2018).

### Case Study 1: AKT1 Inhibitors

**Figure 5:**
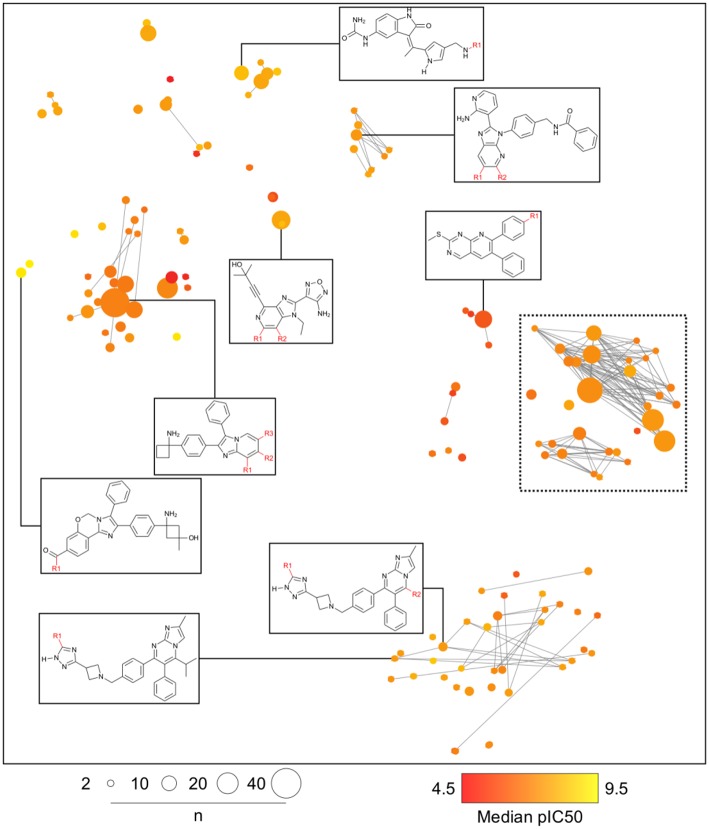
General constellation plot for a dataset of AKT1 inhibitors. It is possible to navigate this map, as observed in Figure 6, where the constellation framed within dashed lines is further explored.

Analogs in this library could be summarized in 144 cores as discussed in the section Methods. The cores were organized in 79 analog series and contained 440 compounds (about half of the initial dataset). Figure 5 is the constellation plot for these data, where it becomes apparent that chemical space and chemical substructure information play simultaneous roles in describing the SAR. For instance, although some inactive cores are close to active cores in chemical space, they are not usually contained in the same analog series. Therefore, these could be categorized as “scaffold cliffs” rather than simple activity cliffs conceptualized as two small molecules with similar structures and very different activities (Maggiora, 2006). In this case, collections of molecules, rather than single molecules, are being compared.

**Figure 6:**
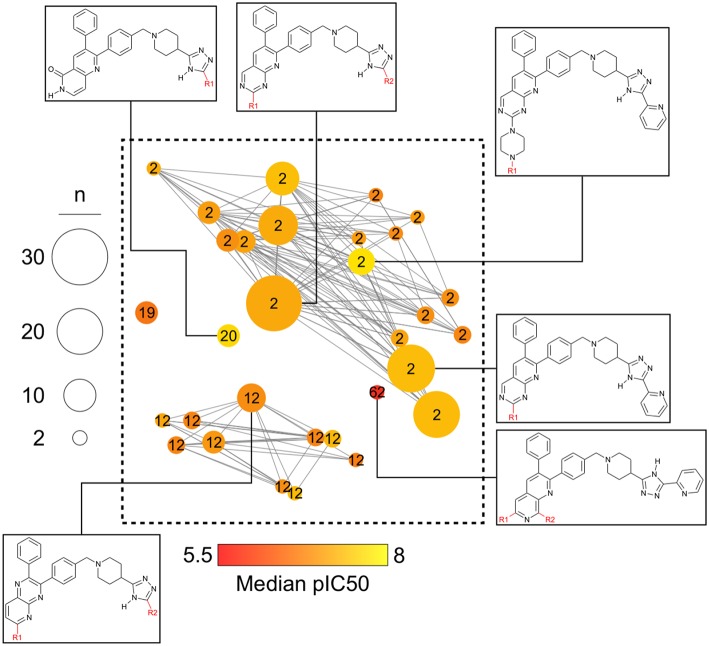
“Zoom-in” into the constellation plot for AKT1 inhibitors selected from Figure 5. The core in analog series 62 is not as active as the nearby cores in analog series 2 and 12. Few structural differences can be noted for the compounds in this constellation.

Figure 6 is a zoomed-in picture into a single “bright” (or predominantly active) constellation comprising five analog series and 55 compounds. As it is readily observed, analog series close in the chemical space have only slight dissimilarities within their scaffolds; in this case, they all share a naphthyridine or naphthyridinone scaffold. Constellation plots allow for a more precise visual SAR analysis and generation of hypotheses. For instance, the core associated to analog series 62 has only a different position for the nitrogens in the rings as well as where substitutions occur. Structural studies could then be conducted to elucidate which are the most relevant features for this kind of scaffolds to be active against AKT1. In this regard, a recent publication co-crystallizing 1,6-naphthyridinone derivatives similar to those in analog series 20 has shown that this scaffold is relevant in forming a π-π stacking interaction with the side chain of Trp80 of the PH-domain (Uhlenbrock et al., 2019). Nonetheless, variation of the position of nitrogen atoms in the scaffold were not considered in the cited study. Indeed, previous SAR studies of these analogs have found the position of the nitrogen atoms in these scaffolds to be critical for the activity against AKT (Zhao et al., 2005;Bilodeau et al., 2008).

### Case Study 2: DNMT Inhibitors

**Figure 7:**
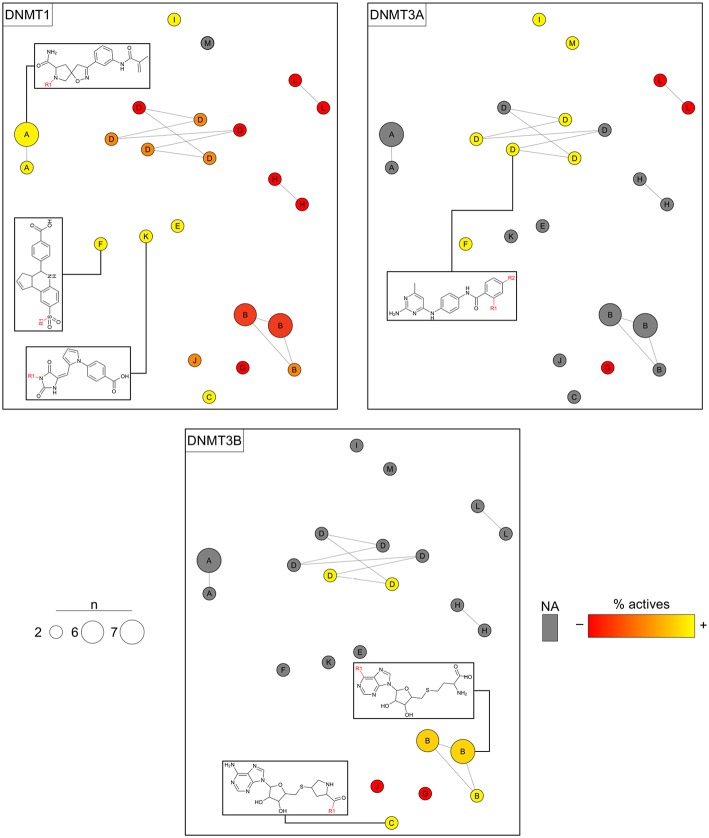
Constellation plots for a dataset of DNA methyltransferase (DNMT) inhibitors tested with DNMT1, DNMT3A, and DNMT3B.

Analogs in this library could be summarized in 23 cores following the procedure discussed in the section Methods. The cores were organized in 13 analog series and contained 46 compounds (about 16% of the initial dataset). Compounds in this library have annotated activity with DNMT1, DNMT3A, and/or DNMT3B. Figure 7 shows three constellations plots, where chemical space is the same and colors change to represent the activities against each DNMT. As elaborated on the section Methods, each circle in the plot represents a core in which coordinates in the 2D graph is given by similarity measurements computed from Morgan fingerprints using *t*-SNE for dimensionality reduction. Labels indicate the analog series to which cores belong. The color represents the percentage of active compounds sharing that core using a continuous color scale from red (less active cores) to yellow (more active cores). For this example of use of the constellation plots, the definition of “active” was determined from integrating qualitative and quantitative data sources as described in Naveja and Medina-Franco (2018). Circles in gray indicate cores with no reported activity for that particular DNMT. The size of the circle indicates the number of compounds sharing the core. Connected circles are cores sharing compounds. Figure 7 also shows the chemical structures of representative cores.

The constellation plots for DNMT inhibitors in Figure 7 allow for rapidly getting several interesting insights of the SAR. For instance, cores at the top left part of the plot from analog series “A” are a bright constellation against DNMT1, i.e., a region in chemical space with active analogs. However, these analogs have not been tested against the other DNMT isoenzymes, which would help determine whether these inhibitors are selective.

Of note, there is a “dark” (or predominantly inactive) constellation in the chemical space of DNMT1 formed by six cores from analog series “D.” This dark constellation, however, is more active overall against DNMT3A and appears to be active against DNMT3B. Furthermore, not all cores in this constellation have been tested against DNMT3A and DNMT3B, where they have greater chances of being active.

The plot also reveals a constellation of nucleoside analogs from series “B” at the bottom-right region of the plot that is, overall, selective toward DNMT3B vs. DNMT1. This series has not been tested against DNMT3A yet. Moreover, most of the cores have been tested in DNMT1 only, thus hindering discussions on selectivity. In this regard, analysis of constellation plots is visually helpful in guiding multitarget drug discovery campaigns and in finding opportunities for selectivity.

## Conclusions and Perspectives

We introduced a novel approach for combining chemical space and analog series methodologies into a single descriptive analysis that can be summarized in a constellation plot. Adding the analog series concept into the chemical space facilitates discussions of regions in the space, as every point summarizes a collection of analogs. A so-called “constellation in chemical space” can be conceptualized as those regions in chemical space formed by core scaffolds with similar structure (as defined by a coordinate-based projection). Mapping activity on the plot readily uncovers active and inactive zones, e.g., bright or dark regions, in chemical space. Of note, constellation plots would be useful for exploring virtually any chemical property, such as biological activity (as demonstrated with two case studies), but also physicochemical properties, complexity or selectivity statistics. In this regard, constellation plots are a flexible approach with multiple potential applications in academia and industry, aiding in the quest of finding potential leads from large collections of screening data (e.g., such as that produced by high-throughput screening campaigns). One of the next steps of this work is the application of the constellations plots to navigate through cell selectivity data of a comprehensive screening dataset. Results will be disclosed in due course.

## Data Availability

The datasets generated for this study can be found in Supplementary Information.

## Author Contributions

JN participated in the conceptualization, data gathering, data analysis, and drafted the first version of the manuscript. JM-F participated in the conceptualization, data analysis, and revision of the manuscript.

### Conflict of Interest Statement

The authors declare that the research was conducted in the absence of any commercial or financial relationships that could be construed as a potential conflict of interest.
