# Supplementary material for: Finding Constellations in Chemical Space Through Core Analysis
Source: Front Chem. 2019 Jul 16;7:510. doi: 10.3389/fchem.2019.00510 (PMC6646408; doi:10.3389/fchem.2019.00510)
Supplement: Supplementary file 1 [file Table_1.DOCX]

SUPPLEMENTARY INFORMATION

**Finding constellations in chemical space through core analysis**

J. Jesús Naveja^1,2*^, José L. Medina-Franco^2,*^

^1^ PECEM, Faculty of Medicine, Universidad Nacional Autónoma de México, Avenida Universidad 3000, Mexico City 04510, Mexico.

^2^ Department of Pharmacy, School of Chemistry, Universidad Nacional Autónoma de México, Avenida Universidad 3000, Mexico City 04510, Mexico.

** Corresponding authors:* [*naveja@comunidad.unam.m*](mailto:navejaromero@gmail.com)*x (JJN),* [*medinajl@unam.mx*](mailto:medinajl@unam.mx) *(JLMF)*

Supplementary_Information.zip contains the following supplementary material of the above-indicated manuscript:

-Jupyter Notebook “constellation-plots.ipynb” contains the relevant code for generating constellation plots in an interactive code live environment. All necessary data to run the notebook is provided within the zip file.

- Python program “get-cores.py” that can be run from a Linux terminal in an RDkit Anaconda environment. Run the following in a terminal to get information about the required arguments: python get-cores.py --help

-Folder “data” with the following files:

-akt.tsv A tab-separated file with the curated AKT1 inhibitors database as described in the main text

-DNMT.tsv A tab-separated file with the curated DNMT inhibitors database as described in the main text

-*ASM.tsv, *ASW.tsv, *cores.tsv Each dataset of the above was preprocessed with “get-cores.py” to extract cores information and generate the files for constellation plots.

-Folder “scripts”: this folder contains relevant scripts used by “get-cores.py” to generate cores. Do not modify files in this folder. Always run get-cores.py in a folder containing the scripts folder as well.
